# Safety and Clinical Response to Combined Immunotherapy with Autologous iNKT Cells and PD-1^+^CD8^+^ T Cells in Patients Failing First-line Chemotherapy in Stage IV Pancreatic Cancer

**DOI:** 10.1158/2767-9764.CRC-23-0137

**Published:** 2023-06-07

**Authors:** Jing Wang, Xiaobo Cheng, Yanling Jin, Bili Xia, Ran Qin, Wei Zhang, Huiliang Hu, Xiaoting Mao, Liting Zhou, Jia Yan, Xiaoyan Zhang, Jianqing Xu

**Affiliations:** 1 Shanghai Public Health Clinical Center, Fudan University, Shanghai, P.R. China.; 2 Clinical Research Center, Obstetrics and Gynecology Hospital of Fudan University, Shanghai, P.R. China.; 3 Shanghai Public Health Clinical Center and Institutes of Biomedical Sciences, Shanghai Medical College, Fudan University, Shanghai, P.R. China.; 4 Clinical Center for Biotherapy, Zhongshan Hospital (Xiamen), Fudan University, Xiamen, P.R. China.; 5 Clinical Center for Biotherapy, Zhongshan Hospital, Fudan University, Shanghai, P.R. China.

## Abstract

**Purpose:**

A phase I clinical trial was conducted to assess the safety and feasibility of invariant natural killer T (iNKT) cells combined with PD-1^+^CD8^+^ T cells in patients with advanced pancreatic cancer and failing the first-line chemotherapy.

**Patients and Methods:**

Fifteen eligible patients were enrolled, of whom 9 received at least three cycles of treatment each. In total, 59 courses were administered.

**Results:**

Fever was the most common adverse event, peaking at about 2–4 hours after cell infusion and reverting within 24 hours without treatment in all patients. Influenza-like reactions such as headache, myalgia, and arthralgia were also observed in 4, 4, and 3 of the patients, respectively. In addition, vomiting and dizziness were prevalent, while abdominal pain, chest pain, rash, and stuffy nose were rare adverse events, each reported in 1 patient. Side effects above grade 2 were not observed. Two patients achieved partial regression, while 1 patient experienced disease progression assessed 4 weeks after the third course. Three patients are still alive at the time of writing and have progression-free survival longer than 12 months. The overall survival time has been extended to over 12 months in 6 of the 9 patients. No constant changes of CD4^+^ T, B, and NK cells were recorded except for elevated CD8^+^ T cells after the first course.

**Conclusions:**

The combination of autologous iNKT cells and PD-1^+^CD8^+^ T cells was a safe therapeutic strategy against advanced pancreatic cancer. The patients exhibited a potentially promising prolonged survival time. Further study appears warranted to evaluate the efficacy of these combined cell infusions in pancreatic cancer.

**Trial registration:**

This trial was included in the clinical trial which was registered in ClinicalTrials.gov (ID:NCT03093688) on March 15, 2017.

**Significance:**

There is an unmet need for novel, more effective, and tolerable therapies for pancreatic cancer. Here we present a phase I clinical trial employing iNKT cells combined with PD-1^+^CD8^+^ T cells in 9 patients with advanced pancreatic cancer and failing the first-line chemotherapy. The combined immunotherapy was shown to be feasible in the enrolled patients with limited side effects and optimistic clinical responses, which could bring opportunity of therapeutic advancement.

## Introduction

Pancreatic adenocarcinoma is the seventh most common cause of cancer-related mortality worldwide and is characterized by an extremely poor prognosis. Patients diagnosed with metastatic pancreatic cancer face a 5-year survival rate of 2.9% (1, 2), and the only available treatments, surgery, chemotherapy, and chemoradiation, have shown limited clinical effectiveness (3). Only 20% of these patients are eligible for surgery with curative intent, and most will develop recurrent disease within 2 years of definitive therapy (4). Therapeutic options for patients with advanced pancreatic cancer failing the first-line chemotherapy are limited. Only ≤50% of patients who fail first-line treatment are still physically fit enough to be offered second-line treatment, for whom the median survival time is rendered less than 7 months (2, 5, 6). Therefore, alternative treatment strategies are urgently needed to improve the prognosis and prolong the survival time. As new therapeutic choices, immunotherapy and targeted systemic therapy can offer improved prognoses for patients with cancer and are under extensive investigation in patients suffering from pancreatic cancer.

Adoptive T-cell transfer (ACT) has emerged as a powerful therapeutic option for liquids (7, 8) as well as solid cancers (9, 10). Administration of tumor-infiltrating lymphocytes (TIL) that can be expanded *in vitro* from a surgically resected tumor provides a particularly promising approach. This therapy for patients with metastatic melanoma was reported to be associated with a 20% complete response lasting beyond 3 years (11). However, the application of TIL infusion relies on the successful isolation and expansion of TILs from the surgically resected tissue. Although *in vitro* expansion of TIL from human pancreatic tumors has proved achievable (11), for the majority of patients surgery was not recommended, either because of metastatic disease or because an incomplete resection (R2) could have a detrimental effect on survival (12–14). Studies from Rosenberg and colleagues performed in patients with melanoma and ovarian cancer have shown that expression of PD-1 on CD8^+^ T cells in circulating blood identified the repertoire of tumor-reactive cells, suggesting that the CD8^+^PD-1^+^ T cells in peripheral blood might be isolated and expanded to provide a surrogate of TILs for use in ACT (15–17). Recently, Li and colleagues demonstrated that T cells expanded from PD-1^+^ peripheral blood lymphocytes, compared with their PD-1^−^ counterparts, share more T-cell receptor (TCR) clones with paired TILs by comparing the TCR repertoire of these three populations, providing further evidence for the tumor reactivity of PD-1^+^CD8^+^ T cells in circulating blood (18). The primary concern and challenge for the adoptive transfer of CD8^+^PD-1^+^ T cells is the potential exhaustion and functionally impaired status of these cells. However, CD8^+^PD-1^+^ T cells can recover antitumor function after culture in an appropriate medium, and improve clinical benefits in advanced melanoma (17, 19, 20).

Because pancreatic cancer is considered immunologically “cold” with a highly immunosuppressive tumor microenvironment (TME), which decreases the functional ability of infiltrated T cells, many studies focused on making tumors immunologically active by applying dendritic cell (DC) vaccines or cytokines along with ACT (20). iNKT cells are known as a subset of lipid and glycolipid-reactive T lymphocytes coexpressing markers of NK cells. Activated iNKT cells can mediate antitumor immunity by directly lysing tumor cells, and indirectly enhancing the antitumor function of other effector cells through secreting IFNγ, TNF, and IL2 (21–23). Furthermore, iNKT cells are regarded as playing essential roles in the regulation of immune suppression in the TME by reducing the frequency and inhibitory function of tumor-associated macrophages (TAM) and myeloid-derived suppressor cells, which are the primary immunosuppressive cells in TME (23–25). A high count of tumor-infiltrated iNKT cells was shown to be associated with a good prognosis in pancreatic adenocarcinoma (26). Even though circulating iNKT cells are functionally defective, and decreased in number in patients with malignant tumors (27, 28), it has been reported that iNKT cells could reinvigorate the secretion of multiple cytokines and cytotoxicity, and iNKT cell–based immunotherapy has seen great preclinical success (23, 29, 30). In addition, iNKT cell–based combined ACT has shown appealing prospects in clinical trials. With or without α-GalCer–pulsed matured monocyte-derived DCs, adoptive infusion of iNKT cells supported immunologic and objective clinical responses in several clinical trials (31–33). Recently, we also reported the reinvigoration of anti-CD20 antibody with the combination of iNKT cells in treating an human immunodeficiency virus (HIV)-infected patient with diffuse large B-cell lymphoma. Pathologically complete regression was induced, lasting over 42 months till now (34).

We proposed that the adoptive transfer of iNKT cells combined with PD-1^+^CD8^+^ T cells would potentiate the clinical response by the reinforcement of the antitumor function of both cells and the consequent regulation of the TME by iNKT cells. The work of Delfanti and colleagues highlighted the antitumor efficacy of iNKT cells combined with tumor-specific T cells in mice which was superior to the separate infusion of iNKT cells and tumor-specific T cells (35). We conducted a clinical trial to evaluate the safety and feasibility of autologous iNKT cells combined with PD-1^+^CD8^+^ T cells expanded *ex vivo* in clinical treatment of patients with diverse various solid tumors (ClinicalTrials.gov ID: NCT03093688). In previous studies, we reported the preliminary data of safety and feasibility of the combined regimen of iNKT cells and PD-1^+^CD8^+^ T cells in patients with lung adenocarcinoma (36). In this study, we reported for the first time the clinical trial, making use of the integrated approach with iNKT and PD-1^+^CD8^+^ T cells to treat patients with pancreatic cancers that had failed first-line chemotherapy, which was included in NCT03093688, with the primary endpoint of safety, and secondary endpoints of response rate and survival.

## Materials and Methods

### Patients

This trial was a single-arm, prospective, single-center phase I clinical trial aiming to explore the safety and feasibility of combined immunotherapy of iNKT and PD-1^+^CD8^+^ T cells in patients with pancreatic cancer. It was conducted in accordance with Declaration of Helsinki, and approved by the institutional local ethical committee of Shanghai Public Health Clinical Center [Institurional Review Board number: (2017)2017-S005-01]. All participants provided written informed consent before study entry.

All enrolled patients had advanced metastatic pancreatic cancer with pathologic diagnosis verified by imaging within 30 days before treatment initiation, had failed the first-line chemotherapy, with a Karnofsky performance status (KPS) score no less than 80, were ages between 18 and 75 years and had a life expectancy >3 months. Patients had not received chemotherapy in the 4 weeks preceding the start of treatment. Exclusion criteria included: a history of immunodeficiency disease or autoimmune disease; organ dysfunction; lymphoma or leukemia; a bleeding disorder; myelodysplastic syndrome; severe infections that need antibiotic treatment; positive for HIV antibodies, hepatitis B surface antigen or hepatitis C virus RNA; within concurrent chemotherapy, pregnancy or lactation; participated in a study of an investigational agent within 4 weeks before the first dose of the study intervention.

### Treatment Schedule

**FIGURE 1:**
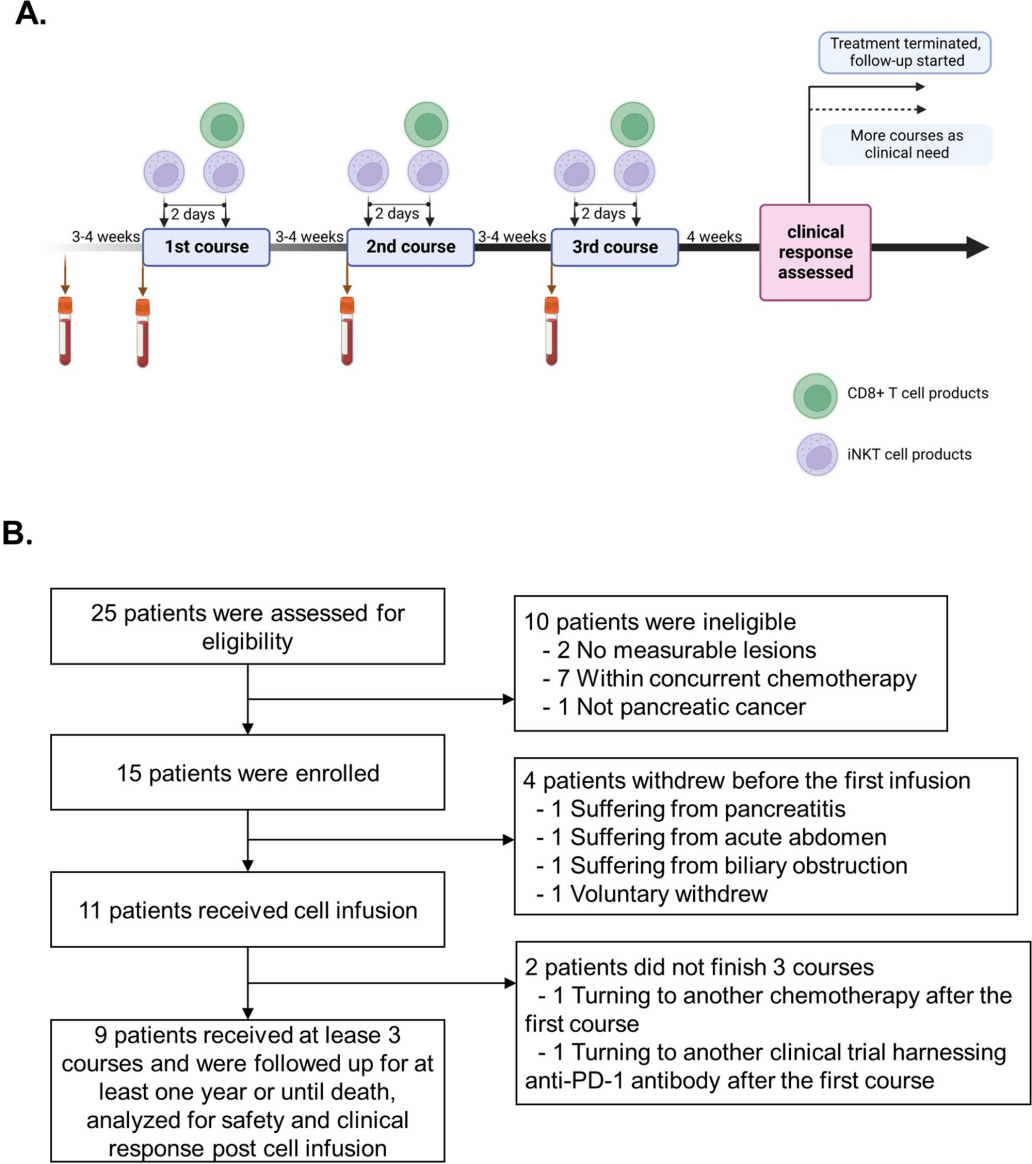
Schematic of study design and patient deposition. **A,** Treatment regimen. **B,** Patient deposition per Consolidated Standards of Reporting Trials guidelines.

A scheme of the study design is shown in Fig. 1A. All patients received at least three cycles of treatment. Every 4–5 weeks, patients received one infusion solely of iNKT cells and one infusion 2 days later of a combination of iNKT and PD-1^+^CD8^+^ T cells. Autologous iNKT-enriched cells (2 × 10^8^ to 3 × 10^9^) were intravenously infused on the first day and autologous iNKT cells (2 × 10^8^ to 3 × 10^9^) mixed with PD-1^+^CD8^+^ T cells (2 × 10^7^ to 2 × 10^9^) were transfused on the third day during each course. Extensive clinical and laboratory assessments were conducted in every course and consisted of a complete physical examination and standard laboratory assessments. Adverse events were graded according to the NCI Common Terminology Criteria for Adverse Events V.3.0 (CTCAE). Patients were followed up for at least 1 year or until death.

### PD-1^+^ CD8^+^ T-cell Isolation and *Ex Vivo* Expansion

Isolation and expansion of PD-1^+^CD8^+^ T cells were performed following the method we published previously (37). Briefly, for isolation of blood-derived PD-1^+^CD8^+^ T cells, peripheral blood mononuclear cells (PBMC) were first isolated and, after staining with CD3-BV421 (BD Pharmingen), CD8-APC-H7 (BD Pharmingen), and PD-1-PE-CF594 (BD Pharmingen), they were sorted into 96-U-well cell plates at 5,000 cells per well using a FACS Aria II cell sorter (BD Biosciences, certificated medical device by Chinese National Medical products administration). All the following procedures after cell sorting were performed in a GMP-like cleaning room. The cells were allowed to settle for 1 hour before the medium was replaced with fresh X-VIVO 15 culture medium (Lonza), to which anti-CD3/anti-CD28 beads (cells:beads = 1:1) were added together with recombinant human IL7 (R&D, 20 ng/mL), recombinant human IL15 (R&D, GMP grade, 20 ng/mL), Pam3CSK4 (InvivoGen, 100 ng/mL), FSL-1 (InvivoGen, 10 ng/mL), and flagellin (InvivoGen, 10 ng/mL). After 7-day incubation, the cells were transferred to a 24-well plate for further expansion under the same culturing/stimulation conditions and then to a T25 flask and larger flasks at appropriate timepoints according to the cell growth rate. The final cell yields were harvested between 21 and 28 days after the initial seeding.

### Expansion of iNKT Cells *Ex Vivo*

All procedures were performed in a GMP-like cleaning room. iNKT cells were cultured *in vitro* following the method described in our previous work (38). Briefly, PBMCs (1 × 10^7^) were seeded into 6-well plates with 100 ng/mL KRN7000 (Funakoshi) in the X-VIVO (Lonza) culture medium and then cultured at 37°C in a humidified atmosphere containing 5% CO_2_ for 1 week. We separately added recombinant human IL7 (20 ng/mL; R &D, GMP grade, 207-GMP-01M) and IL2 (100 IU/mL; R&D, GMP grade, 202-GMP-01M) to the culture medium on the sixth day. Autologous DCs loaded with KRN7000 were added to the culture medium every week. Recombinant human IL15 (20 ng/mL, R&D, GMP grade, 247-GMP-01M) was added 1 week before harvest, and IL12 (20 ng/mL, R&D, GMP grade, 219-GMP-01M) was added to the culture medium one day before harvest. All the cells were harvested, washed four times, resuspended in 250 mL of normal saline solution, and ready for infusion.

### Release Criteria of Expanded Cells

Sterility, viability, purity, and cytokine release were assessed as quality controls during the culture period and before harvest. Sterility testing was performed to ensure that the endotoxin level was ≤0.5 EU/mL and there was no *Mycoplasma* contamination. The viability and purity of cells were assessed by using flow cytometry. Viability was detected by using Annexin V PE Apoptosis Kit (BD) and the percentage of Annexin V and PE double-negative cells was higher than 90%. The percentage of iNKT cells was identified as CD3^+^Vα24^+^ cells. The percentage of CD8^+^ T cells was used as a quality control standard for PD1^+^CD8^+^ T cells because cells lost expression of PD-1 during proliferation *in vitro*, becoming no less than 95%. The functional activity of the iNKT cell was assessed by determining the cytokine profile (IFNγ and IL4) in the culture medium after the *in vitro* expansion by flow cytometry. The ratio of IFNγ/IL4 should be higher than 500.

### Immune Monitoring

A total of 10 mL of blood was collected in each course before cell infusion and 4 weeks after the last cell administration. Detection of NK, B, and T cells was performed following the instructions of the detection kit (BD Multitest IMK). Plasma and the remaining cells were frozen until further analysis. Th 1/2/17 cytokines in plasma were detected by using cytometric beads array (CBA) according to the instructions of the kit (BD Biosciences, 560484). α-Galcer–responding cells in PBMCs were detected to confirm the impact of iNKT cell infusion using enzyme-linked immunospot assay. Briefly, 2 × 10^5^ PBMCs per well were stimulated in triplicate by α-Galcer at the concentration of 2 μg/mL in 96-well plates precoated with the first anti-IFNγ antibody and incubated for 2 hours. After incubation, the second anti-IFNγ antibody was added, followed by an horseradish peroxidase–conjugated antibody specific to the second antibody. The plates were read after the spots were clearly displayed. The result was recorded as the number of α-Galcer–specific cells per 1 × 10^6^ PBMCs. Amino acid sequences of CDR3 of TCRβ were determined to identify TCRβ repertoire. CD8^+^ T cells in the expanded cells and in PBMCs were sorted using FACS Aria II cell sorter (BD Biosciences), reserved in TRIzol and transported to the ExAb Corporation to perform TCR sequencing.

### Clinical Monitoring

The primary endpoints were safety, assessed according to the “NCI CTCAE” (version 3.0), and objective tumor response, assessed by independent central review according to RECIST (version 1.1). A third key endpoint was overall survival (OS; time from the first dose to death due to any cause).

Radiologic evaluations (CT, PET-CT, or MRI) were performed during the 2 weeks preceding treatment initiation, again 4 weeks after the third cycle, and 4 weeks after every three cycles if the patients received more than three courses according to clinical need. Extensive laboratory testing for tumor markers was conducted in each course of treatment. Patients were followed up after the last cycle for at least 1 year or until death.

### Data Availability Statement

The data generated in this study are not publicly available because the medical records and private data included could compromise patient privacy but are available upon reasonable request from the corresponding author.

## Results

### Characteristics and Treatment of Enrolled Patients

Fifteen eligible patients were enrolled among 20 screened patients. Three patients dropped out before the first infusion because of pancreatitis, acute abdomen, and biliary obstruction, respectively. One patient withdrew voluntarily. One patient decided to receive another chemotherapy after the first cell infusion, and another one patient turned to another clinical trial harnessing anti-PD-1 antibody, both of whom were excluded from the analysis (Fig. 1B). Nine enrolled patients received at least three cycles of treatment in this clinical trial, and were included in the analysis, among whom 3 were ages between 50 and 59, another 3 were 60–69 and the other 3 were 70–74. Five of the patients were female. All had advanced pancreatic cancer with metastasis to sites including lymph nodes, lungs, liver, and adrenal glands. Eight of the patients were pathologically confirmed ductal adenocarcinoma except patient P001 who was diagnosed with sarcomatoid carcinoma (Table 1). Representativeness of study participants were presented in Supplementary Table S1. All patients had failed in systemic first-line chemotherapy that included FOLFIRINOX (5-fluorouracil, leucovorin, irinotecan, and oxaliplatin) for P006, and AG (nab-paclitaxel plus gemcitabine) and S-1 (tegafur, gimeracil, and oteracil) for the other 8 patients.

**TABLE 1:** Patient characteristics, treatment, and OS outcomes

Patient	Age	Gender	Stage at study entry		KPS^a^	Site of disease					Total tumor burden^b^ at study entry (mm)	Previous treatment	Courses received	PFS, months	OS, months
			AJCC	TNM		Pancreas	LN	Lung	Liver	Other visceral					
001	70	F	IV	M1c	80	X	X		X		80.3	S-1, Albumin-bound paclitaxel	15	7	18
002	65	M	IV	M1b	90	X		X			58.8	AG, S-1	13	>47	>47
003	74	M	IV	M1c	90	X			X		136.3	Gemcitabine hydrochloride, S-1	3	–	4
004	58	M	IV	M1c	90	X			X		51.8	S-1, Gemcitabine hydrochloride	3	5	12
005	57	F	IV	M1c	80	X	X		X		79	AG	8	13	25
006	66	M	IV	M1c	90	X		X	X		75	FOLFIRINOX, AG	5	5	8
007	67	F	IV	M1b	90	X	X	X			40	AG	4	>16	>16
008	72	F	IV	M1c	80	X		X	X		42	Albumin-bound paclitaxel, S-1	4	>15	>15
009	50	F	IV	M1c	80	X	X	X		X	51	S-1, AG	4	6.5	12.5

Abbreviations: AG, nab-paclitaxel plus gemcitabine; FOLFIRINOX, 5-fluorouracil, leucovorin, irinotecan, and oxaliplatin; S-1, tegafur/gimeracil/oteracil.

^a^Karnofsky performance status.

^b^Sum of tumor diameters.

A total of of 59 courses and 118 infusions were performed in this study. Of the 9 patients included in this trial, 7 were treated according to protocol and received at least three courses of cell infusions, missing at most one dose of CD8^+^ T cells, except for P001 who received only iNKT cells without CD8^+^ T cells during all infusions because her PD-1^+^CD8^+^ T cells could not be expanded *ex vivo* after being sorted. P003 and P004 received three courses, while the other patients received more cycles as clinical needs (Table 1).

### Infusion Products

Leukapheresis was not applied in our study. Instead, 40 mL peripheral blood was collected into anticoagulant tubes by venipuncture to obtain PBMCs 4 weeks before the first course and each course before the first iNKT cell infusion. iNKT and PD-1^+^CD8^+^ T cells were expanded following the method reported in our previous work (37, 38). Details of the infused cellular products are summarized in Supplementary Table S2.

**FIGURE 2:**
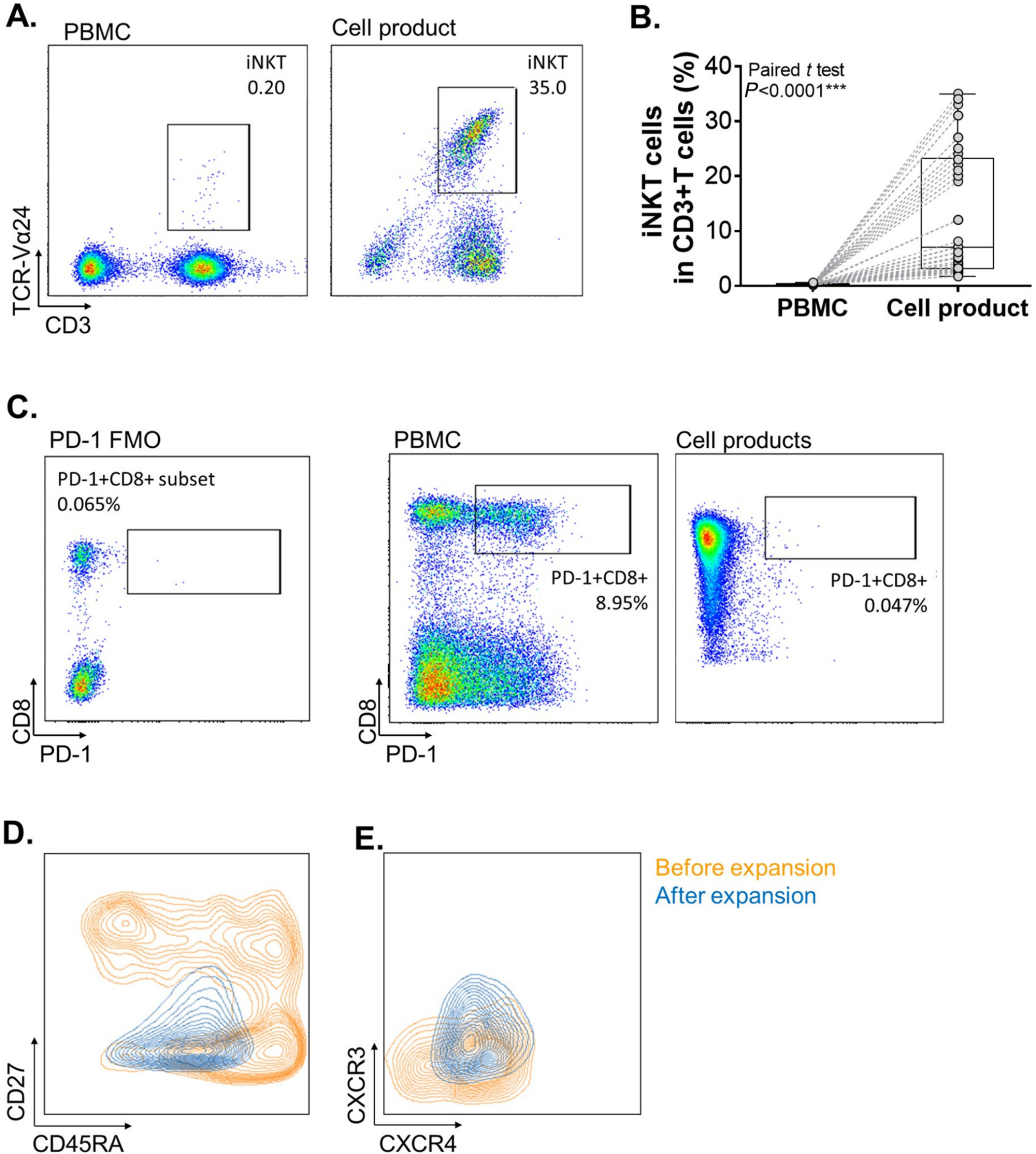
Characteristics of iNKT cells and PD-1^+^CD8^+^ T cells in the cell products were assessed by flow cytometry. **A,** Gating strategy of iNKT cells identified as CD3^+^TCR Vα24^+^ cells in PBMCs (left) and in cell products after expansion (right). **B,** The purity of iNKT cells as a percentage in CD3^+^ T cells before and after expansion *ex vivo* (paired *t* test). **C,** Gating strategy of PD-1^+^CD8^+^ T cells. PD-1 FMO was applied to rigorously define the PD-1^+^ cells. **D,** Memory status of PD-1^+^CD8^+^ T cells before (orange) and after expansion *ex vivo* (blue) by staining CD27 and CD45RA on cell membrane. **E,** Staining of CXCR3 and CXCR4 on PD-1^+^CD8^+^ T cells before (orange) and after expansion (blue).

The iNKT cells were identified as CD3^+^TCR Vα24^+^ cells by flow cytometry, and the percentage of iNKT cells in T cells prior to expansion was verified as 0.01%–0.2% (39). Expansion *ex vivo* resulted in a marked enrichment (Fig. 2A and B); the purity of iNKT cells postexpansion varied from 5% to 65% (median 12%) and the total number of iNKT cell products ranged from 2.5 × 10^8^ to 3.9 × 10^9^ (median 2.3 × 10^9^). The ratio of IFNγ/IL4 in the supernatant before cell harvest was considered as a functional index of iNKT cells (40) and varied from 700 to 25,323.

The percentage of PD-1^+^CD8^+^ T cells in T cells was 0%–10% and could be enriched to 95% after expansion *in vitro*. Because some expression of PD-1 was lost during the process of culturing and the percentage of PD-1^+^ cells in the product was less than 5% (Fig. 2C), the purity of CD8^+^ T cells in the cell products was assessed as releasing criteria instead of PD-1^+^CD8^+^ T cells and was higher than 95%. The number of CD8^+^ T cells infused ranged from 1.27 × 10^7^ to 2.7 × 10^9^ (median 6.55 × 10^8^). Some deviations of the infusion regimen were included when PD-1^+^CD8^+^ T could not be expanded successfully from the limited numbers (less than 10,000 cells after sorting by flow cytometry in some instances). PD-1^+^CD8^+^ T cells were not included in the cell infusions in one course of P002, P003, P006, and P007, and in all courses of P001.

Our preclinical study on the composition and function of PD-1^+^CD8^+^ T-cell products suggested that the expanded CD8^+^ T cells expressed low levels of checkpoint ligands, CTLA-4 and PD-1, and high levels of CD107a, granzyme B and IFNγ, which excluded the concern about the potential exhaustion of such population (37). Herein, we applied flow cytometry to verify the composition of PD-1^+^CD8**^+^** T cells preexpansion and postexpansion only for several enrolled patients. Infused CD8^+^ T cells were mainly CD27-CD45RA^low^ cells which were regarded as effector memory cells (Fig. 2D). High levels of ICOS and 4-1BB, and low levels of CTLA-1 and PD-1 were expressed on the expanded CD8^+^ T cells (Supplementary Fig. S1A and S1B). CXCR4 and CXCR3 were regarded to play important role on the homing and infiltration of T cells to the tumor sites (41), and were elevated on expanded PD-1^+^CD8**^+^** T cells (Fig. 2E; Supplementary Fig. S1C and S1D).

### Clinical Observations

**FIGURE 3:**
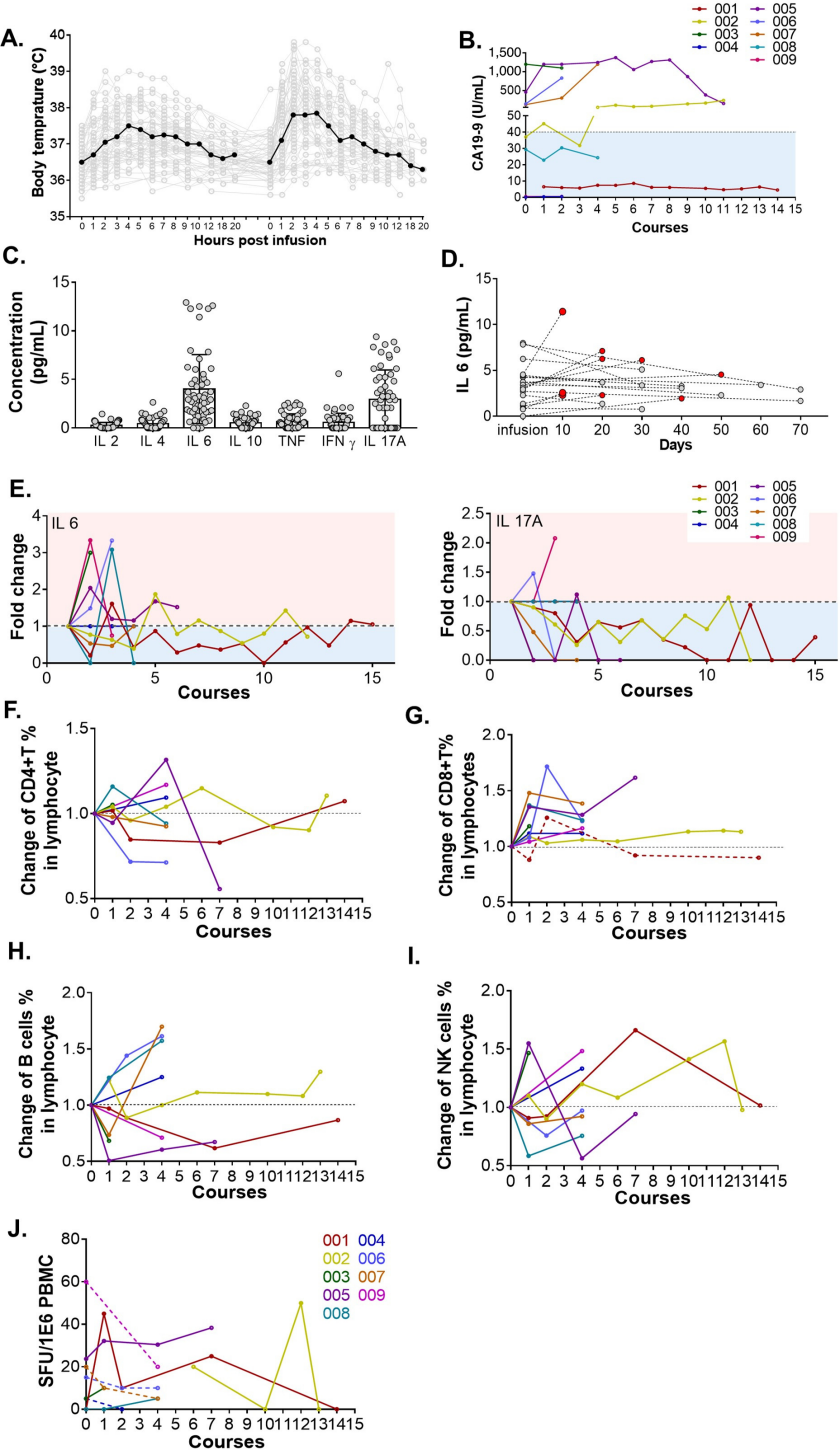
Clinical records and detections. **A,** The body temperature of patients recorded each hour for 20 hours after cell infusion. All the records in 59 courses are included here in gray. The mean temperature in each timepoint is shown in black bold. Changes in tumor markers CA19-9 in serum during cellular therapy were shown in **B**. Impact of cell infusion on the cytokines in serum and key populations of lymphocytes in PBMCs. Quantification of Th1/2/17 cytokines in plasma after cell infusion were analyzed by CBA. All results are displayed in **C**. Change of IL6 in serum over time are shown in **D**, on which red dots indicated elevated IL6 levels post infusion. Change of IL6 (left) and IL17A (right) during treatment are shown in **E**. Changing percentage of CD4^+^ T, CD8^+^ T, B, and NK cells during treatment are assessed by flow cytometry, and data relative to baseline are shown in **F**, **G**, **H**, and **I**, respectively. Changes in number of IFNγ-secreting cells responding to α-Galcer were also recorded using ELISPOT and are shown in **J** with increased IFNγ-secreting cells responding to α-Galcer in some patients (solid) and not in others (dotted).

The combination of iNKT and CD8^+^ T cells was safely administered to all patients. All patients experienced fever and chill after cell infusion, no matter whether this was solely iNKT cells for the first infusion or the combination of the two cell populations in the second infusion. However, the time intervals between the cell infusion and the increase in body temperature were shorter and the peaks were higher during the second infusion in each course. The peak of the body temperature appeared about 4–5 hours after the first infusion and 2–4 hours after the second infusion (Fig. 3A). Body temperatures recovered in 20–24 hours without treatment. No other grade 2 adverse events were observed except for nine grade 2 fevers recorded in 59 courses. Influenza-like reactions, such as headache, myalgia, and arthralgia, were the second most common side effects observed in 4, 4, and 3 of the 9 patients, respectively. Even though vomiting was observed only in four of 59 courses, it appeared in 3 of 9 patients and was regarded as prevalent in the studied patients, which was the same with dizziness and nausea. On the contrary, even though abdominal pain was recorded six times in 59 courses, it was reported by only 1 patient and rendered as a relatively rare side effect. Chest pain, rash, and stuffy nose were also rare adverse events (Table 2).

**TABLE 2:** Adverse events

Adverse events	Grade 1, *n*	Grade 2, *n*	Grade 3, *n*	Grade 4, *n*	Grade 5, *n*	Total, *n*	Occurrence rate (all patients)
Dizziness	3	0	0	0	0	3	2/9
Abdominal pain	6	0	0	0	0	6	1/9
Chills	26	0	0	0	0	26	9/9
Fever	39	9	0	0	0	48	9/9
Nausea	2	0	0	0	0	2	2/9
Vomiting	4	0	0	0	0	4	3/9
Headache	10	0	0	0	0	10	4/9
Arthralgia	8	0	0	0	0	8	3/9
Myalgia	7	0	0	0	0	7	4/9
Chest pain – cardiac	1	0	0	0	0	1	1/9
Rash maculopapular	1	0	0	0	0	1	1/9
Stuffy nose	1	0	0	0	0	1	1/9
**Total**	108	9	0	0	0	117	

A series of tumor markers were assessed over time in each course (Fig. 3B; Supplementary Fig. S2) including CA19-9 which is regarded to be substantially associated with the progress of pancreatic cancer (Fig. 3B). Nevertheless, CA19-9 was constantly normal in 2 of total 9 patients, patient 001 and 004, for whom no abnormal tumor marker was observed. Although the abnormal tumor markers differ among the enrolled patients, changes of abnormal tumor markers make sense in portending progress of the tumor. In our clinical trial, the raising of the originally elevated tumor marker and the emergence of new abnormal tumor markers are always observed to be associated with the disease progress.

### Immune Monitoring

Immune monitoring was applied to evaluate the engraftment of infused cells and the impact of cell infusion on other cell populations in the circulating blood. Blood samples for immune monitoring were taken in each cycle prior to the first iNKT cell infusion and 4 weeks after the last treatment. Th 1/2/17 cytokines in plasma were assessed over time during treatment. The concentration of all the cytokines detected, including IL2, IL4, IL6, IL10, TNF, IFNγ, and IL17A, are low in the summarized data, among which IL6 and IL17A are relatively higher (Fig. 3C). IL6 was shown to be elevated in plasma taken at the timepoint close to the cell infusion (Fig. 3D). Even though higher in concentration than the other cytokines detected, none of IL6 and IL17A was cumulatively elevated during treatment (Fig. 3E).

The detection of frequency of CD4^+^ T, CD8^+^ T, B, and NK cells in PBMCs by flow cytometry and α-Galcer–specific cells by ELISPOT were also applied (Fig. 3F–I). The gating strategy in an individual case is shown in Supplementary Fig. S3A. The frequency of CD8^+^ T cells was moderately increased after the first course in the 8 patients who received the CD8^+^ T-cell infusion, and remained elevated relative to baseline in the following cycles, except for patient 001 who did not receive the CD8^+^ T cells (Fig. 3G). In addition, deep sequencing on CDR3 of TCRβ of CD8^+^ T cells was performed in the reserved cell products and their paired PBMCs 4 weeks after infusion in 3 patients, patient 002, 003, and 007. More than 20% of top 50 TCR clones of infused CD8^+^ T cells were readily identifiable in PBMCs, especially the top 10 TCR clones of which ≥50% were identifiable in PBMCs (Supplementary Fig. S4), which suggests the potential engraftment of infused CD8^+^ T cells together with the constant elevated frequency of CD8^+^ T cells after infusion. Although fluctuations were observed in the quantity of CD4^+^ T, B, and NK cells, no consistent change was noted during the treatment process in the patients overall (Fig. 3F, H, and I; Supplementary Fig. S3). The impact of infused iNKT cells on the frequency and activation state of the other cell populations was reported to last for a week or two after infusions before returning to approximately baseline levels (42). In our study, the interval between bleedings was longer at 4 weeks and the transient effects of infused iNKT cells and CD8^+^ T cells on the other cell populations would not be detected.

iNKT cells account for a small fraction of PBMCs. Changes in the frequency of iNKT cells in PBMC, that are slight, would be difficult to assess by flowcytometry based on the tissue-homing character of the cells (43). We detected IFNγ secreting cells specific to stimulation with α-Galcer instead. The count of α-Galcer–specific cells was noted to be increased compared with the baseline level on at least one occasion in 5 patients, while no increase was noted in the other 4 patients (Fig. 3J). Besides, the proliferation speed of iNKT cells during expansion *ex vivo* was observed to be improved over time under the comparation of the cell growth curve in each course, which potentially suggested the engraftment of infused iNKT cells and the invigoration of the endogenous iNKT cells, especially in patient 001 and patient 002 who received more than five courses (Supplementary Fig. S5).

### Clinical Outcomes

**FIGURE 4:**
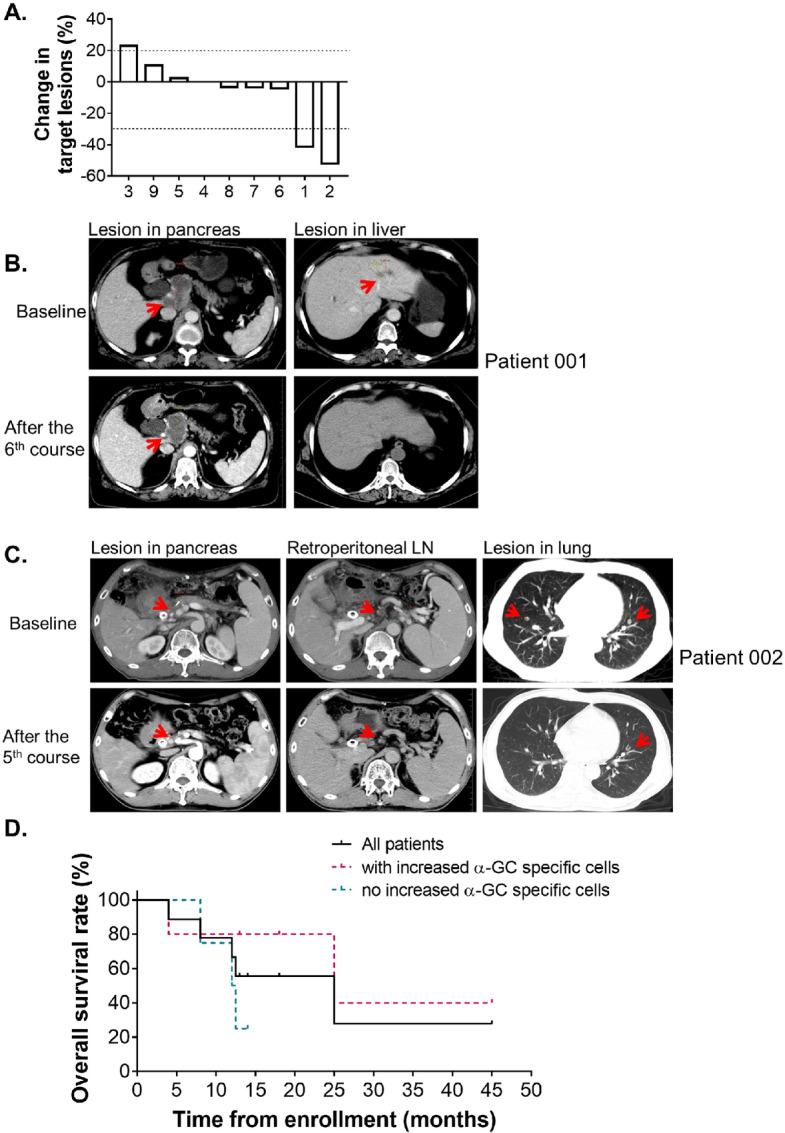
Clinical response and OS data. **A,** Waterfall plot showing best overall change in sum of diameter of tumor lesions for each patient. Change in sum of tumor diameters by RECIST is compared with baseline. Representative CT images of patient 001 and patient 002 with PR of the sum of lesions are shown in **B** and **C**, respectively. **D,** Kaplan–Meier estimate of OS of all 9 patients (black), patients with elevated IFNγ-secreting cells responding to α-Galcer (pink) and the others (blue).

Change in sum of tumor diameters by RECIST is compared with baseline. The size of the study does not allow conclusions about the clinical efficacy of the treatment. However, it is interesting to report that of all 9 analyzed patients, patient 001 and 002 achieved partial regression (PR) at best during treatment (Fig. 4A). PR was achieved in patient 001 4 weeks after the third cycle and lasted for 7 months. The patient 001 died of acute respiratory failure after an influenza infection in the 19th month. Patient 002 experienced a PR after the fifth course, which is maintained at 30 months after the last treatment. Patient 003 had 23% enlargement in the sum of total diameters at the first CT scan performed 4 weeks after the third course, though the other 6 patients maintained stable disease which lasted for 5 to 16 months. (Fig. 4A).

Besides the partial remission of the target lesion in the pancreas, one of the metastatic lesions was completely regressed in patient 001 and 002, respectively. The two metastatic lesions in the liver of patient 001 were regressed at the first CT scan performed 4 weeks after the third course, and were confirmed to be completely regressed on the CT scan 4 weeks after the sixth course, which was maintained in the last CT scan 15 months after enrollment (Fig. 4B). One of the metastatic lesions in the lung of patient 002 was completely remised 4 weeks after the fifth course (Fig. 4C).

All patients were followed up for 4 to 45 months to clarify the prognosis and cause of death. The OS time of 6 of the patients has been prolonged beyond 12 months (Table 1). The 1-year survival rate was 66.7% (Fig. 4D). Even though not statistically significant, patients with increased α-Galcer–responsive cells showed moderately better OS (Fig. 4D).

## Discussion

Despite the promising advances in drugs and treatments over the last several decades, pancreatic cancer is still a disease that poorly responds to conventional therapies and there have been no major improvements in survival. With promising potential, ACT is now being tested for treating advanced tumors and the efficacy of chimeric antigen receptor (CAR)-T against pancreatic cancer has been evaluated in clinical trials (44). However, CAR-T has provided limited benefit in pancreatic ductal adenocarcinoma (PDAC) so far (45). The complexity of TME, the stromal hindrance that limits immune response, and the expression of checkpoint blockade by T cells all pose hurdles, just as reported in the treatment of other kinds of solid tumors. By acting as immunoregulatory cells, iNKT cells play an indispensably important role in the antitumor immune response by enhancing the survival and effector activities of tumor-specific cells. By cytolyzing TAM, they decrease the suppressive microenvironment besides direct cytotoxic function against tumor cells. Therefore, active iNKT cells might be used therapeutically, either alone or in combination with other treatments, to reverse the defects in iNKT cells, improve the antitumor immune response, and achieve a better prognosis (46, 47).

PD-1^+^CD8^+^ T cells in peripheral blood were identified to be a patient-specific tumor-reactive CD8^+^ lymphocyte population and proposed to perform in the same way as TIL (15). PD-1 was used as a marker identifying the tumor-specific CD8^+^ T cells with TCRs recognizing tumor cells. We have reported an efficient method to expand PD-1^+^CD8^+^ T cells *ex vivo* by using ligands of Toll-like receptors as costimulators (37). Interestingly, we found that the PD-1 expression was downregulated during the expansion yielding a fraction of PD-1^+^ cells in CD8^+^ T cells less than 5%. However, the expanded cells were proposed to sustain the tumor specificity of TCR on the CD8^+^ T cells in the cell products. An extensive study on the detailed characteristics of TCR repertoire during *in vitro* expansion was reported by Li and colleagues (18). Nevertheless, no direct proof of tumor recognition by the T-cell products could be provided in our study with no tumor samples obtained from the enrolled patients who had previously experienced several treatments including surgery. Regarding the possible mechanisms of tumor regression in patient 002, potential tumor-specific recognition and depletion by infused PD-1^+^CD8^+^ T cells could not be ruled out for the complete remission of the metastatic lesion and regression of local lesion in the pancreas, which would be regarded as auxiliary evidence. Modified design and sample collection to explore the tumor recognition by the cell products are warranted in the further clinical study.

iNKT cells and tumor-specific T cells in patients with cancer were reported to be functionally impaired compared with their counterparts without cancer, and the malfunctions exacerbated along with the progress of disease (48–50). iNKT can be reinvigorated to secret Th1 cytokines after *ex vivo* expansion (39, 51, 52) and in this study, although the purity of iNKT cells in the cell product varied among different patients, the ratio of IFNγ/IL4 was consistently higher than 500. It is worth noting that some infusions were performed without PD-1^+^CD8^+^ T-cell products due to the failed expansion *ex vivo* in this study. On the basis of the fact that sorting the PBMCs that were isolated from only 10 mL blood yielded only 5,000 to 20,000 PD-1^+^CD8^+^ T cells, one explanation for the failed expansion is that the low count of initial cells seeded mounted the difficulty of cell expansion. For the patient P001, we were unable to expand the PD-1^+^CD8^+^ T cells for the first three courses but succeed in yielding 2 × 10^7^ CD8^+^ T-cell products for the fourth cycle. Nevertheless, she achieved PR as assessed 4 weeks after the third course, so CD8^+^ T cells were not added in the following courses to evaluate the progression-free survival (PFS) of this 1 patient under sole iNKT cell immunotherapy.

We chose to collect anticoagulated blood by venipuncture for each course instead of performing leukapheresis before the first cycle to simplify the procedure of cell collection and further reduce the potential harm to the patient. Moreover, the TCR repertoire of tumor-specific CD8^+^ T cells could evolve along with the evolution of the tumor cells and disease progression. Hence, compared with those at the baseline, PD-1^+^CD8^+^ T cells isolated after each cycle could differ a lot in TCR repertoire and be more efficient with better tumor specificity. Lymphodepleting chemotherapy preconditioning is regarded to be pivotal to improve ACT therapy efficacy by eliminating endogenous lymphocytes that compete for homeostatic cytokines, including IL2 and IL7, which are require for the engraftment and persistence of infused cells (53), and reducing regulatory T cells (Treg) which in turn strengthen the antitumor efficiency of infused cells (54), but is associated with significant toxicities of febrile neutropenia (55). Higher dose of lymphodepleting chemotherapy was reported to be associated with increased incidence rate of febrile neutropenia compared with low dose, but were not significantly associated with higher objective responses (55). In our clinical trial, according to the fact that all 9 patients received chemotherapy prior to the study entry, of whom peripheral cytopenia was noted, lymphodepleting chemotherapy was not performed so as to avoid potential severe toxicity. More than three consecutive courses were applied as compensation to enhance the persistency of infused cells.

The primary goal of our study was to validate the safety and feasibility of the adoptive transfer of autologous iNKT cells consolidated with PD-1^+^CD8^+^ T cells expanded *in vitro* as a potential treatment for patients with pancreatic cancer. Given the complicated disease status and treatment choices, the drop-out rate of this study is high. Among the 15 enrolled patients, only 9 received at least three cycles each, and were included in the analysis. Although the small scale of our study did not allow us to draw conclusions regarding the clinical efficacy of the combined infusion of autologous iNKT cells and PD-1^+^CD8^+^ T cells, 58 cycles were performed in total providing with substantial clinical records and detections for preliminarily analyzing the safety of the combined infusion. Most adverse events were grade 1 and only nine grade 2 fevers were recorded. Consistent with the observations of previous clinical trials using ACT, transitory fever was the most prevalent adverse event observed in 39 of 59 infusions in this study, occurring from 2 hours after cell infusion and resolving without treatment within 20 to 24 hours. There were no signs of dose-induced cytokine storm or GVHD.

It is promising that the survival time of 6 of the 9 patients extended beyond 12 months, exceeding the life expectancy of patients who received only the second-line chemotherapy. Notably, 2 patients, P001 and P002, achieved PR at best during treatment. The patient P001 was diagnosed as sarcomatoid carcinoma pathologically and was resistant to the first-line therapy of AS (Albumin-bound paclitaxel and S-1) before study entry. She was the only enrolled patient who received solely iNKT cells. During the treatment, PR was achieved with complete remission of the metastases in the liver for 7 months till the appearance of new metastases inside the abdominal wall. Unfortunately, she suddenly died of acute respiratory failure caused by influenza infection in the 19th month. iNKT cells were deduced to be essentially associated with the clinical response in patient 001, while the role of CD8^+^ T cells was uncertain in any of the patients. Schalck and colleagues have clarified the landscape of TIL in patients with pancreatic cancer, which provides a framework with subpopulations besides PD-1–positive cells on which to modify future immunotherapies using tumor-specific effector cells (56).

In addition, the densely fibrotic tumor microenvironment, caused by cancer-associated fibroblasts and desmoplasia, together with the immunosuppressive tumor environments are characteristically seen in pancreatic cancer and impedes the antitumor effect of infiltrated immunocytes (57). To our knowledge, there is no evidence of the combined cell regimen employed in this clinical trial to overcome the stomal barrier is reported. Prominent fibrosis is a histopathologic hallmark of PDAC and often occupies 40%–80% of the total tumor area (58), which is a prominent reason for the resistant to the antitumor treatment. The patients with tumors in which blood vessels exist close to tumor cells, are fortunately expected to benefit from the cell infusion; while patients with tumors in which blood vessels are distanced from tumor cells by stomal tissue, would receive combined treatment of modulation of the fibrotic stroma, such as stomal ablation, with antitumor therapeutics.

The patient P003 had an oversized tumor burden of 136 mm before cell infusion, which may have contributed to poor responsiveness. Enormous tumor burden was revealed to be related to poor prognosis and inadequate efficacy of immunotherapy in patients with liposarcoma and advanced non–small cell lung cancer (59, 60). A total lesion diameter ≥76 mm was independently associated with poor PFS (60). Most patients in this study had a tumor lesion smaller than 85 mm in diameter except P003. Endeavors aiming to reduce the tumor burden were valuable to be pursued to increase the probability of clinical efficacy.

The interval between cell infusion and immune monitoring was 4 weeks in this study, with blood collected before the first infusion in each cycle. Frequent blood sampling after cell adoption was overruled by the Ethics Committee. No consistent changes in CD4^+^ T, B, and NK cells were recorded except for the rise of CD8^+^ T cells after the first course. Hence, the impact of cell infusion on the other cell populations in circulating blood was not clearly described in this clinical trial. Exley and colleagues reported that the change of cell populations following expanded iNKT cell infusion lasted for 1 to 2 weeks, including an elevation of activated NK cells (42). Thus, the potential change of populations in peripheral blood might be concealed by the inappropriate long interval of 4 weeks, which lead to the result of no clear correlation between outcome and immune parameters in this study. Assessment of cell population changes in circulating blood at 2 weeks after administration of cell therapy is more rational.

Several major limitations in our study need to be replenished in further studies. First, even though the elevated frequency of α-galcer–responsive cells and CD8^+^ T cells in lymphocytes were noted during treatment, the engraftment of the infused cells was not clarified with direct evidence because of the failed distinguishment of the administered cells from their endogenous counterparts. Modified design is warranted in future studies. On one hand, deep sequencing of TCRβ would be performed to track the infused CD8^+^ T cells in PBMCs proinfusion and postinfusion. On the other hand, Bluestone and colleagues had tracked the adoptively transferred expanded polyclonal Tregs by metabolic labeling of cells with [6,6-2H2] glucose (61), providing potential methods for safely assessing the engraftment of infused cells without genetic modifications. Second, tumor recognitions of iNKT and CD8^+^ T cells could be directly proved by detecting the response of expanded cells to the lysates of resected tumor tissues. Third, the data from the 9 enrolled patients were limited for drawing convincing conclusions. Clinical trial on a larger scale is warranted to detail the safety and efficacy of iNKT cell infusion combined with PD-1^+^CD8^+^ T cells. In addition, although assessment of cell population changes in circulating blood is important in evaluating safety after cell administration, the capacity of cells for chemotaxis to infiltrate the tumor lesion is essential for antitumor functions. More endeavors should be directed toward the detection of infused cells in the tumor site.

Herein, we present the first report of a clinical trial using iNKT cells combined with PD-1^+^CD8^+^ T cells as a therapeutic strategy against pancreatic cancer. It was promising that the cell combination was feasible for safe use in such a patient population, and 2 patients achieved PR at best. Further studies are merited to investigate the clinical efficacy of the combination of autologous iNKT cells and PD-1^+^CD8^+^ T cells and associated relevant factors in treating patients with pancreatic cancer.

## Supplementary Material

Supplementary Table S1 Representativeness of Study Participants

Supplementary Table S2 Count of CD8+T cell and iNKT cell products in each course

Supplementary Figure S1 Expression of costimulatory receptor (A. ICOS and 4-1BB) and checkpoint ligands (B. CTLA-4 and PD-1) are assessed in expanded PD-1+CD8+ T cell products (n=5). Expression of CXCR4 and CXCR3 on PD-1+CD8+ T cells pre- and post-expansion are assessed by flow cytometry, and shown in C and D respectively (n=5).

Supplementary Figure S2 Change of tumor markers recorded over time during treatment.

Supplementary Figure S3 Gating strategy to detect CD4+ T, CD8+ T, B and NK cells by flow cytometry. Only living cells were included in the analysis (A). Changing in percentages of CD4+ T, CD8+ T, B, and NK cells during treatment are shown in panels B, C, D and E respectively.

Supplementary Figure S4 Heatmap shows the frequencies of the top 50 TCR clones in CD8+T cells in cell products pre-infusion and PBMCs four weeks post infusion. Scale bar, %.

Supplementary Figure S5 Proliferation curve of iNKT cells of P001 (A) and P002 (B) during expansion ex vivo. Curves representing iNKT cells expanded in later courses is shown in darker color.
