# Supplementary material for: Safety and Clinical Response to Combined Immunotherapy with Autologous iNKT Cells and PD-1+CD8+ T Cells in Patients Failing First-line Chemotherapy in Stage IV Pancreatic Cancer
Source: Cancer Res Commun. 2023 Jun 7;3(6):991–1003. doi: 10.1158/2767-9764.CRC-23-0137 (PMC10246506; doi:10.1158/2767-9764.CRC-23-0137)
Supplement: Supplementary Table S1 — Representativeness of Study Participants [file crc-23-0137-s01.docx]

| **Supplementary Table S1.** **Representativeness of Study Participants** | | |
| --- | --- | --- |
| **Cancer type(s)/subtype(s)/stage(s)/condition** | | Pancreatic cancer in stage IV |
| **Considerations related to:** | | |
| **Sex** | Male sex is one of the risk factors of this malignant disease. Male/female incidence ratio: 1.3/1.0. | |
| **Age** | Both 89.4% of new cases of pancreatic cancer and 92.6% of deaths occur in patients over 55 years of age in the United States, the new cases are most frequently diagnosed among people 65-74 years of age with a median age at diagnosis of 70 years, and the percent of deaths is also highest among people of the same age group with a median age at death of 72 years. | |
| **Race/ethnicity** | Striking differences in the frequency of pancreatic cancer exist between races, with rates in the black population being appreciably higher than in white people, and rates are lowest in some Asian populations. | |
| **Geography** | At present, the incidence and mortality of pancreatic cancer are increasing year by year worldwide, no matter in the United States, Europe, Japan, or China. According to Cancer Statistics 2021, the American Cancer Society reported approximately 60430 new cases and 48220 deaths for pancreatic cancer in the United States.  Average age-standardized rates (ASRs) of pancreatic cancer incidence and mortality vary widely across regions of the world. The ASR of the incidence was highest in Eastern Europe, with 9.9 per 100000, followed by Western Europe (9.8), Northern America (9.3), Southern Europe (8.4), Northern Europe (8.3), Australia/New Zealand (7.9), Micronesia/Polynesia (7.7), and Western and Eastern Asia (7.0).  The proportion of estimated new cases for pancreatic cancer in China was relatively high in East China (9.4 per 100000), Northeast (9.4), Northwest (6.8), and North China (5.3), and was comparatively low in Central China (5.2), Southwest (4.3), and South China (3.6), having obvious regional characteristics. | |
| **Other considerations** | Malignant tumors of the pancreases have different histological features i.e., 1) Ductal adenocarcinoma 2) Cystadenocarcinoma and 3) Other (sarcomas, metastatic etc.) malignant tumors. 90% of cases are adenocarcinomas.  Effective screening is not available for PDAC, and most patients present with locally advanced (30%–35%) or metastatic (50%–55%) disease at diagnosis. Survival is significantly better for patients with locally advanced disease (median survival 9–15 months) than for those with metastatic disease (3–6 months). | |
| **Overall representativeness of this study** | The age distribution of our study is similar to that of pancreatic cancer in the literature, median age of 66.  No sex bias of the enrolled patients is included in our study, of whom five in all nine patients are female.  Although our study population was limited to our institution's inpatients in Shanghai, China, the enrolled patients come from 5 different provinces range from north to south in geography. Eight of all nine patients are pathologically confirmed ductal adenocarcinoma, while one patient is diagnosed with sarcomatoid carcinoma. | |
