# Supplementary material for: Safety and Clinical Response to Combined Immunotherapy with Autologous iNKT Cells and PD-1+CD8+ T Cells in Patients Failing First-line Chemotherapy in Stage IV Pancreatic Cancer
Source: Cancer Res Commun. 2023 Jun 7;3(6):991–1003. doi: 10.1158/2767-9764.CRC-23-0137 (PMC10246506; doi:10.1158/2767-9764.CRC-23-0137)
Supplement: Supplementary Table S2 — Count of CD8+T cell and iNKT cell products in each course [file crc-23-0137-s02.docx]

| **Supplementary Table S2. Count of CD8+T cell and iNKT cell products in each course** | | | | | | | | | | | | | | | |
| --- | --- | --- | --- | --- | --- | --- | --- | --- | --- | --- | --- | --- | --- | --- | --- |
|  |  |  |  |  |  |  |  |  |  |  |  |  |  |  |  |
|  | **1** | **2** | **3** | **4** | **5** | **6** | **7** | **8** | **9** | **10** | **11** | **12** | **13** | **14** | **15** |
| **CD8+T cells** | | | | | | | | | | | | | | | |
| 1 | / | / | / | / | / | / | / | / | / | / | / | / | / | / | / |
| 2 | 1.38E+09 | 2.60E+08 | / | 2.20E+07 | 6.50E+07 | 9.69E+08 | 2.00E+08 | 1.18E+09 | 2.25E+08 | 6.22E+08 | 1.17E+09 | 2.70E+09 | 3.21E+08 |  |  |
| 3 | 5.60E+08 | / | 4.00E+07 |  |  |  |  |  |  |  |  |  |  |  |  |
| 4 | 1.94E+09 | 2.00E+09 | 1.13E+09 |  |  |  |  |  |  |  |  |  |  |  |  |
| 5 | 1.31E+09 | 1.18E+09 | 4.05E+08 | 2.10E+08 | 9.66E+08 | 5.87E+07 | 1.27E+07 | 1.36E+07 |  |  |  |  |  |  |  |
| 6 | 1.43E+08 | / | 1.91E+09 | 6.88E+08 |  |  |  |  |  |  |  |  |  |  |  |
| 7 | 1.71E+09 | 6.96E+08 | 9.87E+07 | / |  |  |  |  |  |  |  |  |  |  |  |
| 8 | 2.65E+09 | 1.51E+09 | 1.41E+09 | 2.69E+09 |  |  |  |  |  |  |  |  |  |  |  |
| 9 | 2.24E+07 | 9.44E+07 | 3.21E+07 | 4.07E+07 |  |  |  |  |  |  |  |  |  |  |  |
| **iNKT cells** | | | | | | | | | | | | | | | |
| 1 | 4.50E+08 | 5.60E+08 | 5.20E+08 | 3.50E+08 | 7.66E+08 | 7.29E+08 | 4.56E+08 | 7.90E+08 | 5.70E+08 | 3.10E+09 | 3.00E+09 | 4.70E+09 | 6.70E+09 | 2.30E+09 | 1.76E+09 |
| 2 | 5.10E+08 | 2.60E+09 | 8.20E+08 | 5.90E+08 | 6.00E+09 | 2.90E+09 | 2.80E+09 | 7.00E+08 | 2.30E+09 | 4.20E+09 | 4.50E+09 | 5.70E+09 | 4.00E+09 |  |  |
| 3 | 4.00E+08 | 4.80E+08 | 1.15E+09 |  |  |  |  |  |  |  |  |  |  |  |  |
| 4 | 1.31E+09 | 1.22E+09 | 1.36E+09 |  |  |  |  |  |  |  |  |  |  |  |  |
| 5 | 2.70E+09 | 1.54E+09 | 1.60E+09 | 1.18E+09 | 1.95E+09 | 3.80E+09 | 3.70E+09 | 5.90E+09 |  |  |  |  |  |  |  |
| 6 | 1.41E+09 | 1.40E+09 | 6.00E+09 | 6.25E+09 |  |  |  |  |  |  |  |  |  |  |  |
| 7 | 5.10E+09 | 4.00E+09 | 3.80E+09 | 2.70E+09 |  |  |  |  |  |  |  |  |  |  |  |
| 8 | 2.00E+09 | 2.15E+09 | 3.23E+09 | 3.76E+09 |  |  |  |  |  |  |  |  |  |  |  |
| 9 | 4.70E+09 | 2.50E+09 | 4.40E+09 | 3.20E+09 |  |  |  |  |  |  |  |  |  |  |  |
