# Supplementary figures and images for: Safety and Clinical Response to Combined Immunotherapy with Autologous iNKT Cells and PD-1+CD8+ T Cells in Patients Failing First-line Chemotherapy in Stage IV Pancreatic Cancer
Source: Cancer Res Commun. 2023 Jun 7;3(6):991–1003. doi: 10.1158/2767-9764.CRC-23-0137 (PMC10246506; doi:10.1158/2767-9764.CRC-23-0137)

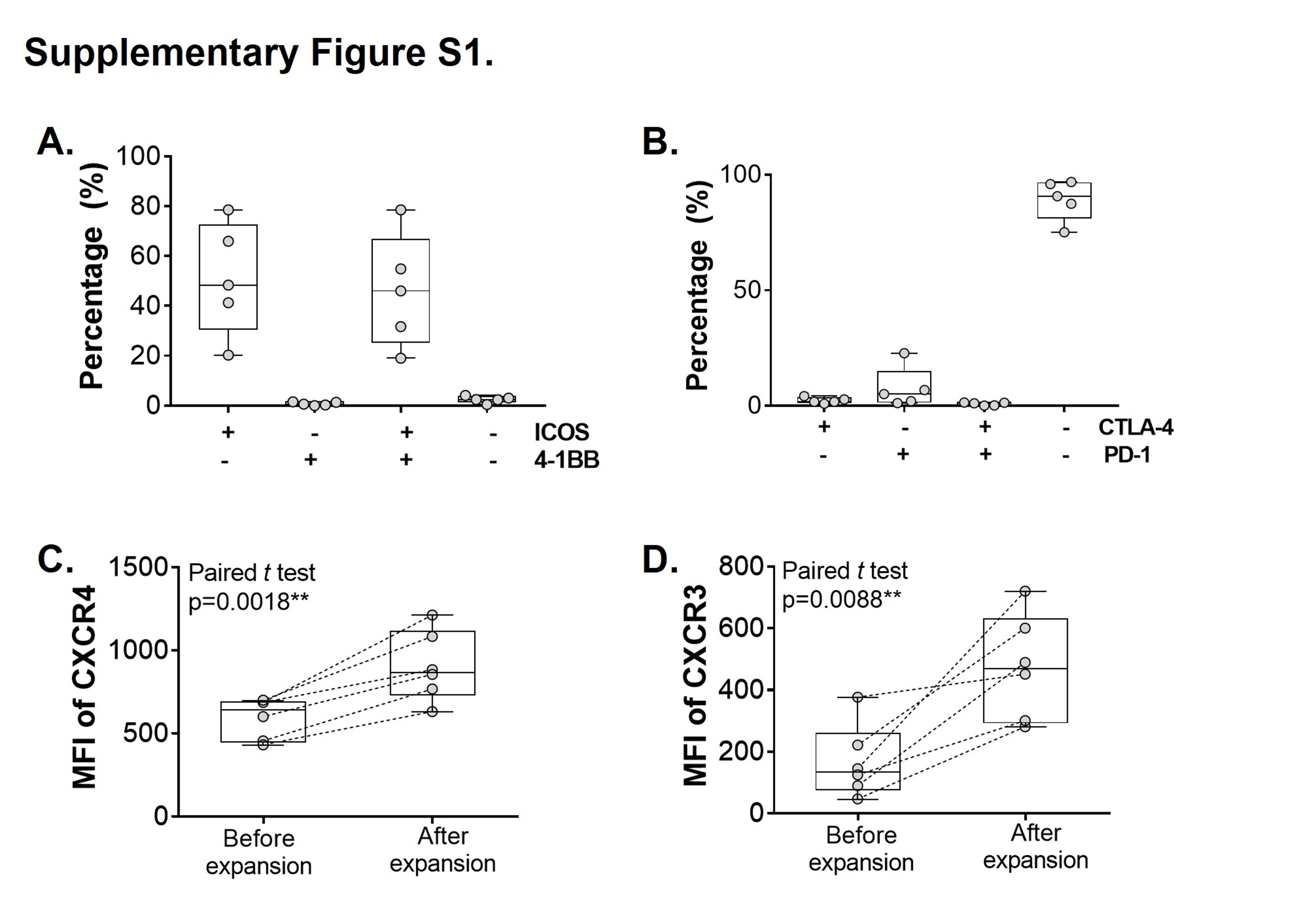

Supplement: Supplementary Figure S1 — Expression of costimulatory receptor (A. ICOS and 4-1BB) and checkpoint ligands (B. CTLA-4 and PD-1) are assessed in expanded PD-1+CD8+ T cell products (n=5). Expression of CXCR4 and CXCR3 on PD-1+CD8+ T cells pre- and post-expansion are assessed by flow cytometry, and shown in C and D respectively (n=5). [file crc-23-0137-s03.png]

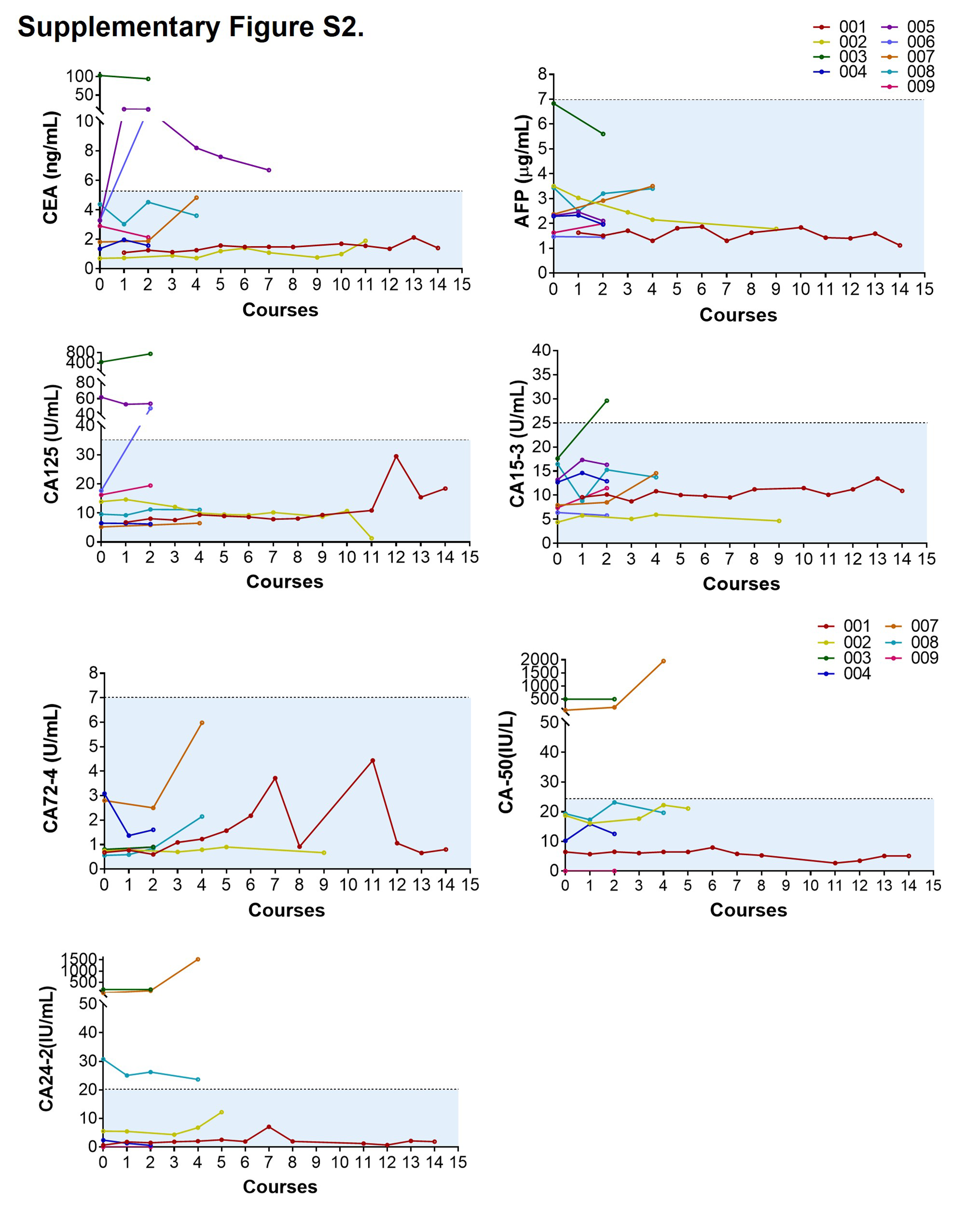

Supplement: Supplementary Figure S2 — Change of tumor markers recorded over time during treatment. [file crc-23-0137-s04.png]

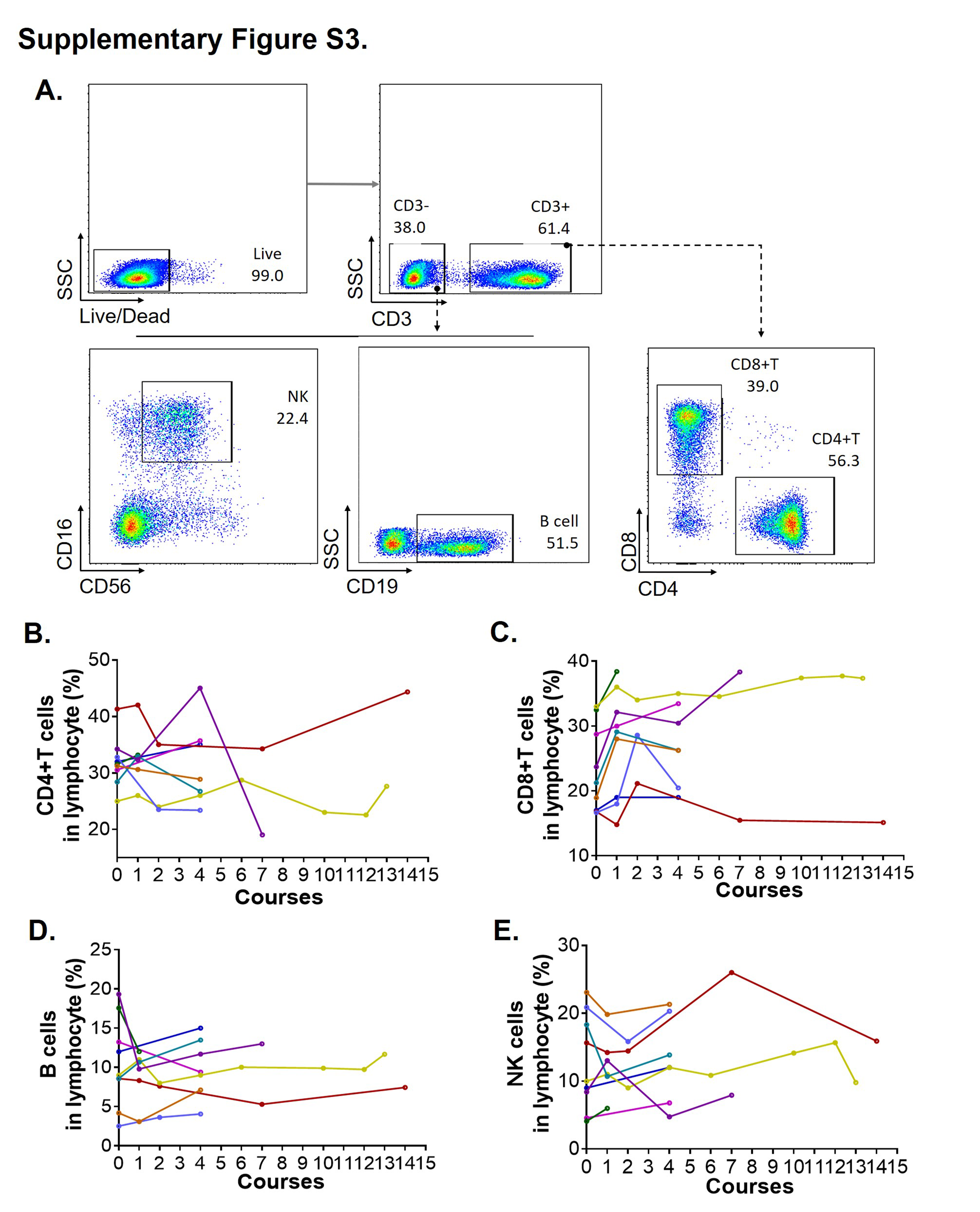

Supplement: Supplementary Figure S3 — Gating strategy to detect CD4+ T, CD8+ T, B and NK cells by flow cytometry. Only living cells were included in the analysis (A). Changing in percentages of CD4+ T, CD8+ T, B, and NK cells during treatment are shown in panels B, C, D and E respectively. [file crc-23-0137-s05.png]

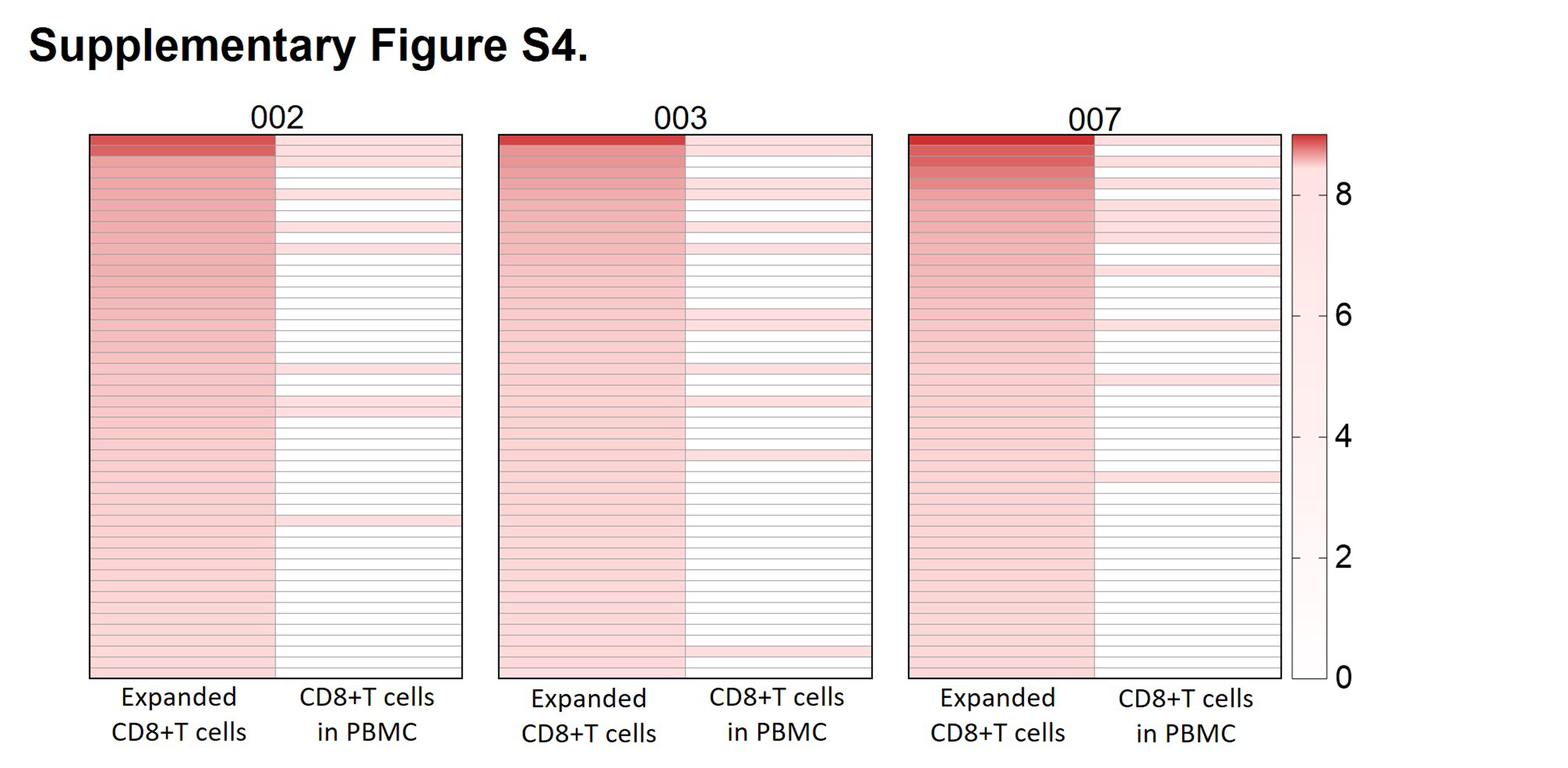

Supplement: Supplementary Figure S4 — Heatmap shows the frequencies of the top 50 TCR clones in CD8+T cells in cell products pre-infusion and PBMCs four weeks post infusion. Scale bar, %. [file crc-23-0137-s06.png]

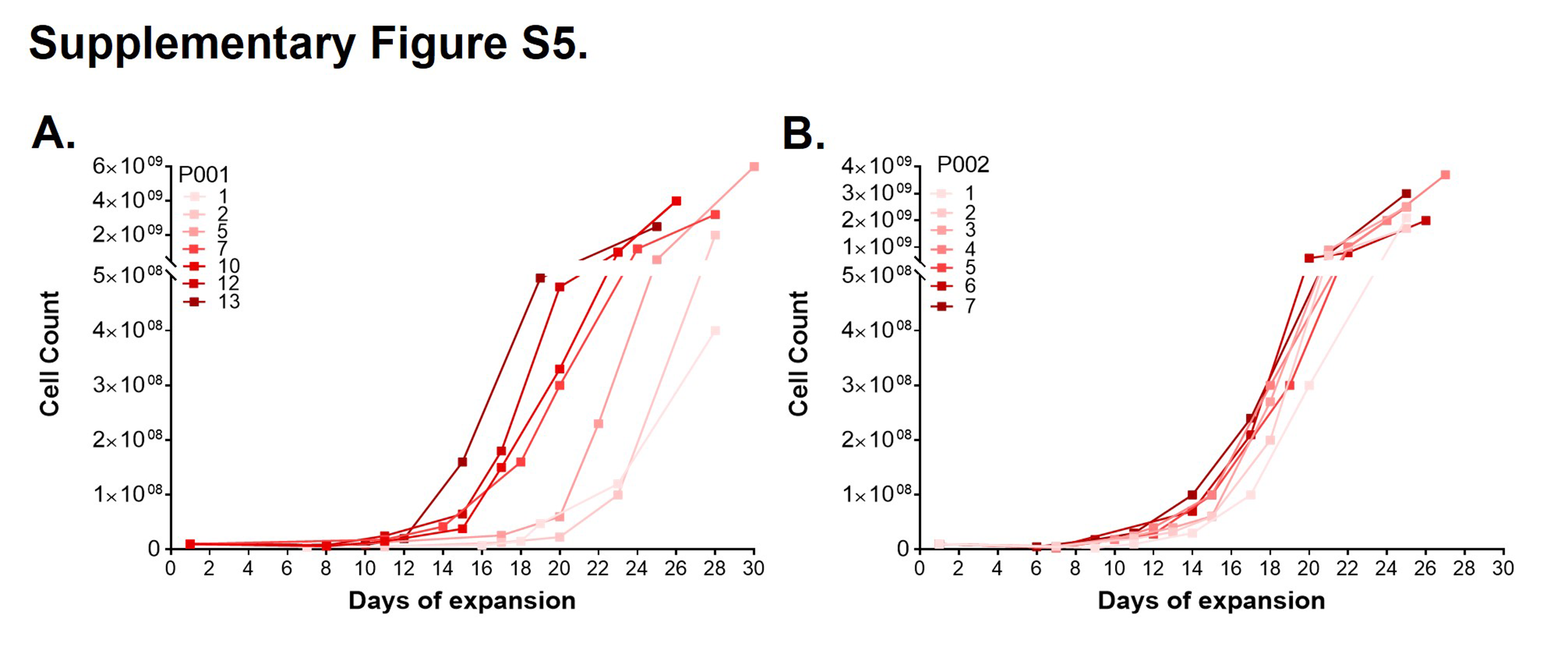

Supplement: Supplementary Figure S5 — Proliferation curve of iNKT cells of P001 (A) and P002 (B) during expansion ex vivo. Curves representing iNKT cells expanded in later courses is shown in darker color. [file crc-23-0137-s07.png]
